# Regional secondary focal segmental glomerulosclerosis in a transplanted kidney – resolution with treatment of a segmental renal artery stenosis

**DOI:** 10.1186/1471-2369-13-38

**Published:** 2012-06-12

**Authors:** Daiki Iwami, Hiroshi Harada, Hiroaki Usubuchi, Kiyohiko Hotta, Toshimori Seki, Masaki Togashi, Yuichiro Fukasawa

**Affiliations:** 1 Department of Kidney Transplant Surgery, Sapporo City General Hospital, 1–1, Kita 11, Nishi 13, Chuo-ku, Sapporo, Hokkaido, 060-8604, Japan; 2 Department of Medical Imaging, Sapporo, Hokkaido, 060-8604, Japan; 3 Department of Urology, Sapporo, Hokkaido, 060-8604, Japan; 4 Department of Pathology, Sapporo City General Hospital, 1–1, Kita 11, Nishi 13, Chuo-ku, Sapporo, Hokkaido, 060-8604, Japan

**Keywords:** Kidney transplantation, Nephrotic syndrome, Renal artery stenosis, Secondary FSGS (focal segmental glomerulosclerosis)

## Abstract

**Background:**

Conditions associated with high intraglomerular filtration pressure can cause secondary focal segmental glomerulosclerosis (FSGS). Unilateral renal artery stenosis (RAS) or its occlusion results in FSGS-like changes and the nephrotic syndrome in the contralateral kidney due to hyperfiltration. However, it has been rarely reported that stenosis of a renal arterial branch can result in FSGS-like changes in a different portion in the same kidney allograft.

**Case presentation:**

A 60-year-old male kidney recipient developed allograft dysfunction after angiotensin II receptor blockade for hypertension 4 months after transplantation. It was proven that one of two arterial branches of the graft was markedly stenotic. Graft dysfunction improved after percutaneous transluminal arterioplasty (PTA), however; the stenosis recurred and massive proteinuria developed 5 months later. Graft biopsy showed ischemic changes in the region fed by the stenotic artery branch and in contrast FSGS-like changes in the region fed by the other branch. His clinicopathological manifestation including massive proteinuria almost normalized after the repeat PTA.

**Conclusion:**

Here we report a case of secondary FSGS of a kidney allograft due to severe RAS of a branch of the same kidney, in which clinical and pathological improvement were confirmed after radiological intervention. When moderate to severe proteinuria appear, secondarily developed FSGS as well as primary (recurrent or de novo) FSGS should be taken into account in kidney transplant recipients.

## Background

Conditions associated with high intraglomerular filtration pressure can cause secondary focal segmental glomerulosclerosis (FSGS) [1,2]. Several groups have reported cases where unilateral renal artery stenosis (RAS) or occlusion results in FSGS-like changes and the nephrotic syndrome (NS) in the contralateral kidney due to hyperfiltration [3-7]. However, it has been rarely reported that stenosis of a renal arterial branch can result in FSGS-like changes in a different portion in the same kidney allograft [8]. Herein, we report a case of secondary FSGS of a kidney allograft due to severe RAS of a branch of the same kidney with pathologically-confirmed improvement after radiological intervention. The study was approved by our Ethical Review Board and conducted in accordance with the Helsinki Declaration.

## Case presentation

A 60-year-old male with end-stage renal disease secondary to IgA nephropathy (biopsy proven), on hemodialysis for 7 years, received a kidney transplant from an ABO-compatible deceased donor outside of Japan (details unknown). Tacrolimus, mycophenolate mofetil, prednisolone and basiliximab were used for induction. He subsequently presented to our institute on postoperative day (POD) 9 with good kidney graft function (serum creatinine, SCr, 0.9 mg/dl without proteinuria). However, on POD 18, he developed elevated SCr and graft biopsy revealed acute T cell-mediated rejection (ATMR, Banff classification, borderline change). He was treated with corticosteroid pulse therapy (500 mg/day of methylprednisolone, for 3 days) and gusperimus hydrochloride (5 mg/kg/day, for 7 days).

**Figure 1:**
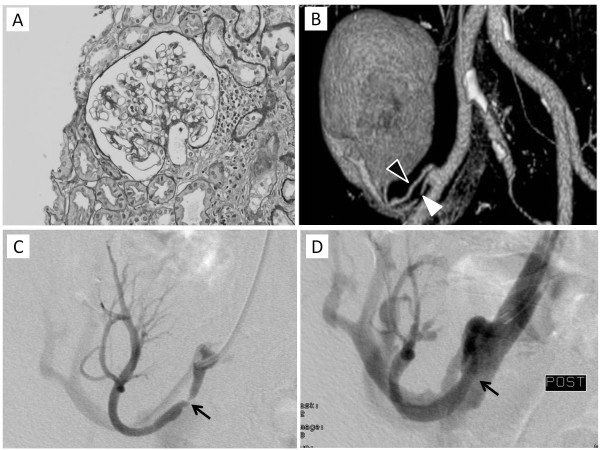
**Findings of kidney allograft biopsy and CT angiography 4 months after kidney transplantation.** (**A**) Kidney graft biopsy showed neither acute rejection nor calcineurin inhibitor nephrotoxicity. (PAS staining original magnification x40). (**B**) CT angiography shows 2 arterial branches (black and white arrowheads) which were reconstructed by conjoined anastomosis at just distal to the anastomotic site on the recipient’s external iliac artery. (**C** and **D**) Percutaneous transluminal arteioplasty successfully dilated the stenotic arterial branch (arrows).

Although he was doing well until POD 118 except for asymptomatic CMV viremia, he suddenly developed severe edema, oliguria, and elevated SCr (from 0.9 to 1.5 mg/dl) after angiotensin II receptor blocker (ARB) treatment was initiated for calcium channel blocker-resistant hypertension. Proteinuria was not severe at that time (urine protein to creatinine ratio, Up/c, 0.41). Kidney graft biopsy demonstrated neither acute rejection nor calcineurin inhibitor nephrotoxicity (Figure 1A). C4d stain was negative on the peritubular capillaries by immunofluorescent stain (data not shown) and anti-donor HLA antibody wasn’t detected by bead-based flow-cytometry assays. A contrast enhanced computed tomography revealed that the kidney graft had two arterial branches (black and white arrowheads, Figure 1B), which were reconstructed by conjoined anastomosis just distal to the external iliac artery anastomosis. One of the branches was markedly stenotic (0.7 mm in diameter, white arrowhead, Figure 1B) and plasma renin activity (PRA) was significantly elevated (31.9 ng/ml/h). After percutaneous transluminal arterioplasty (PTA) of the stenotic branch (Figure 1 C and D), blood pressure (BP) and SCr subsequently returned to baseline.

**Figure 2:**
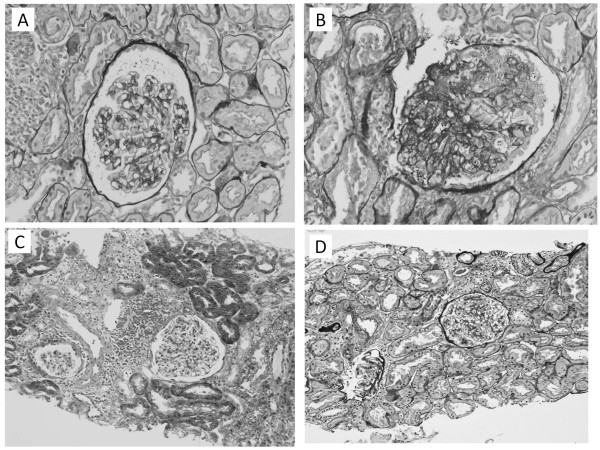
**Pathological findings of kidney allograft biopsy at 9 months (A and B), 14 months (C), and 30 months (D) after kidney transplantation.** (**A**) Ischemic changes are observed in the region of the stenotic arterial branch (PAS staining, original magnification x40). (**B**) Focal segmental glomerulosclerosis (FSGS)-like changes are observed in the region with good blood perfusion (PAS staining, original magnification x40). Allograft biopsy in the well-perfused area performed fourteen (**C**) and thirty (**D**) months after kidney transplantation showed marked improvement of FSGS-like changes by repeated percutaneous transluminal arterioplasty of the stenotic arterial branch (Masson's trichrome staining, original magnification x20).

However, 9 months after kidney transplant, hypertension recurred and massive proteinuria (Up/c, 9.53) developed. Repeat digital subtraction angiography (DSA) showed recurrence of RAS in the same branch. The poorly-perfused and the well-perfused areas of the allograft could be easily distinguished using Doppler ultrasonography. Allograft biopsies were obtained from the two different areas and showed ischemic changes in the poorly-perfused area (Figure 2A) and FSGS-like changes in the well-perfused area (Figure 2B), respectively. Repeat PTA of the stenotic branch was performed with radiographic resolution of the stenosis. Subsequently, the patient’s hypertension and proteinuria markedly improved (Up/c, from 9.53 to 1.80) and the PRA was significantly reduced (from 27.0 to 4.6 ng/ml/h).

Fourteen and thirty months after kidney transplant, respectively, surveillance graft biopsies of the poorly-perfused and well-perfused areas were obtained. Histology of the well-perfused area at 14 and 30 months after the transplant showed significant improvement of the FSGS-like changes (Figure 2 C and D, respectively) while the previously poorly-perfused area demonstrated moderately ischemic changes (Banff classification, interstitial fibrosis and tubular atrophy, IF/TA, grade II, data not shown). The patient currently has excellent graft function (SCr, 0.87 mg/dl) with insignificant proteinuria (Up/c, 0.14), and normal BP without antihypertensive agents 36 months after kidney transplant.

## Conclusions

Secondary FSGS often results in severe proteinuria and can be seen in the setting of several renal diseases. Conditions such as massive obesity, Alport’s syndrome, and uncontrolled hypertension can cause high intraglomerular filtration pressure on the residual nephrons leading to NS [1,2]. As a consequence, adaptive structural changes occur and the remaining glomeruli become sclerotic [1,2]. Renovascular hypertension (RVH) secondary to RAS has previously been shown to cause nephrotic range proteinuria and FSGS-like changes as in the contralateral kidney [4]. Similarly, RVH caused by the stenotic artery branch may have resulted in a high pressure in the glomeruli on the non-ischemic area of the graft in our case. In contrast, it is possible that the area perfused by the stenotic branch may not have been exposed by high pressure which can cause FSGS-like changes. In our case, from the clinical history and medical findings, secondary FSGS caused by stenosis of the arterial branch of kidney allograft was strongly suspected.

In addition, as another possible mechanism, hyperreninemia could cause directly massive proteinuria in our case. From the 1980s, there has been a focus on the relationship between RVH and proteinuiria and hyperreninemia has been implicated as a direct cause of proteinuria of the contralateral kidney with a normal SCr level [9,10] as well as hyperfiltration caused by the secondary hypertension as mentioned above.

Treatment of the basal disorder which causes NS can result in improvement of the proteinuria. In cases of NS caused by RAS, correction of RAS by radiological intervention, surgical repair, or nephrectomy, results in a rapid decrease of urinary protein excretion [10]. However, in kidney transplant recipients, as surgical repair of the stenotic artery is generally difficult and risky, radiological intervention is selected in almost all cases. In our case, repeat PTA successfully diminished proteinuria.and normalized his blood pressure.

In allograft biopsy at 14 and 30 months after the transplant, the affected lesion (perfused by the stenotic artery branch) showed moderate interstitial fibrosis and tubular atrophy. It has also been suggested that ischemia can also cause proteinuria [2]. It is important to follow up carefully to minimize additive potentially harmful causes of kidney allograft damage.

In conclusion, when moderate to severe proteinuria appears in association with hypertension in kidney transplant recipients, secondary FSGS should be taken into account as well as primary (de novo or recurrent) FSGS. As secondary FSGS can be caused by various conditions, it is important to detect and treat the basal diseases which cause the secondary FSGS changes.

## Consent

Written informed consent was obtained from the patient for publication of this case report and any accompanying images. A copy of the written consent is available for review by the Series Editor of this journal.

## Abbreviations

ARB, angiotensin II receptor blocker; ATMR, acute T cell-mediated rejection; BP, blood pressure; DSA, digital subtraction angiography; FSGS, focal segmental glomerulosclerosis; NS, nephrotic syndrome; POD, postoperative day; PRA, plasma rennin activity; PTA, percutaneous transluminal arterioplasty; RAS, renal artery stenosis; SCr, serum creatinine; Up/c, urine protein to creatinine ratio; Hiroshi Harada, Hiroaki Usubuchi, Kiyohiko Hotta, Toshimori Seki, Masaki Togashi, Yuichiro Fukasawa contributed equally to this work.

## Competing interests

The authors declare that they have no competing interests.

## Authors’ contributions

DI, HH, KH, TS, and MT were the treating physicians, HU performed the radiological interventions, and YF performed the evaluation of the kidney allograft biopsy. All of the authors have contributed to the preparation of the manuscript. All the authors have read and agree to the manuscript as written.

## Pre-publication history

The pre-publication history for this paper can be accessed here:

http://www.biomedcentral.com/1471-2369/13/38/prepub
